# Changing Incidence of Uterine Cancer in Rural Egypt: Possible Impact of Nutritional and Epidemiologic Transitions

**DOI:** 10.1200/JGO.18.00255

**Published:** 2019-07-31

**Authors:** Saad Alshahrani, Ahmed Hablas, Robert M. Chamberlain, Jane Meza, Steven Remmenga, Ibrahim A. Seifeldin, Mohamed Ramadan, Amr S. Soliman

**Affiliations:** ^1^ King Fahad Specialist Hospital, Dammam, Saudi Arabia; ^2^ Gharbiah Cancer Society and Gharbiah Population-based Cancer Registry, Tanta, Egypt; ^3^ City University of New York Medical School, New York, NY; ^4^ University of Texas MD Anderson Cancer Center, Houston, TX; ^5^ University of Nebraska Medical Center, Omaha, NE

## Abstract

**PURPOSE:**

Uterine cancer is a top-ranking women’s cancer worldwide, with wide incidence variations across countries and by rural and urban areas. Hormonal exposures and access to health care vary between rural and urban areas, globally. Egypt has an overall low incidence of uterine cancer but variable rural and urban lifestyles. Are there changes in the incidence of uterine cancer in rural and urban areas in middle-income countries such as Egypt? No previous studies have addressed this question from a well-characterized and validated population-based cancer registry resource in middle-income countries. The aim of this study was to explore the differences in clinical and demographic characteristics of uterine cancer over the period of 1999 to 2010 in rural and urban Gharbiah province, Egypt.

**METHODS:**

Data were abstracted for all 660 patients with uterine cancer included in the Gharbiah Population-based Cancer Registry. Clinical variables included tumor location, histopathologic diagnosis, stage, grade, and treatment. Demographic variables included age, rural or urban residence, parity, and occupation. Crude and age-adjusted incidence rates (IRs) and rate ratios by rural or urban residence were calculated.

**RESULTS:**

No significant differences were observed in most clinical and demographic characteristics between rural and urban patients. The age standardized IR (ASR) was 2.5 times higher in urban than in rural areas (6.9 and 2.8 per 100,000 in urban and rural areas, respectively). The rate ratio showed that the IR in urban areas was 2.46 times the rate in rural areas.

**CONCLUSION:**

This study showed that the disease IR in rural areas has increased in the past decade but is still low compared with the incidence in urban areas in Egypt, which did not show a significant increase in incidence. Nutritional transitions, obesity, and epidemiologic and lifestyle changes toward Westernization may have led to IRs increasing more in rural than in urban areas in Egypt. This pattern of increasing incidence in Egypt, which used to have a low incidence of uterine cancer, may appear in other middle-income countries that experience emerging nutritional and epidemiologic transitions. The rate of uterine cancer in urban areas in Gharbiah is almost similar to the corresponding rates globally. However, the rate in rural areas in this population has increased over the past decade but is still lower than the corresponding global rates. Future studies should examine the etiologic factors related to increasing rates in rural areas and quantify the improvement in rural case finding.

## INTRODUCTION

Uterine cancer is among the top-ranking cancers affecting women worldwide.^1^ There are wide international variations in disease incidence and by rural or urban place of residence within countries.^2-5^

Incidence rates (IRs) of cancers, including those for uterine cancer, tend to be higher in urban than in rural populations.^2,6,7^ However, the gap between rural and urban areas in cancer incidence has decreased over time, especially in developed countries.^8^ A limited number of studies exist on uterine cancer distribution by rural and urban areas in developing countries.

Hormonal exposures implicated in uterine cancer etiology and access to health care in disease management vary between rural and urban areas, globally.^3-5,9-12^ According to a study by the Gharbiah Population-based Cancer Registry (GPCR) for 1999 to 2002, the incidence of gynecologic cancers (ovary, uterine, and cervical) was higher in urban than in rural areas.^13-16^ Uterine cancer showed the highest urban–rural incidence rate ratio (IRR) among the three gynecologic cancers (IRR, 6.07; 95% CI, 4.17 to 8.85), whereas ovarian and cervical cancers had IRRs of 2.57 and 3.11, respectively. Urban areas had higher IRs and older age at diagnosis of uterine cancer compared with rural areas and a relatively low rate of uterine cancer in this population compared with the global rates of the disease.^13-15^

The Gharbiah province comprises approximately 5.5% of the Egyptian population, and approximately 70% of the population lives in rural areas.^17^ With the availability of a larger data set from the registry for the period of 1999 to 2010, and the improvement in medical and diagnostic facilities in the region,^18^ this study aimed to examine the rural–urban difference in uterine cancer in Gharbiah.

## METHODS

Data included in this study were obtained by abstracting clinical and demographic information of all 660 patients who resided in the Gharbiah province and were diagnosed with uterine cancer over the period of 1999 to 2010. The GPCR of the Gharbiah province in Egypt was the source of information in this study. The GPCR is a population-based cancer registry established in 1999 by the National Cancer Institute in the United States, through the Middle East Cancer Consortium.^14^ The Gharbiah Cancer Society in Tanta, the capital city of Gharbiah, hosts the GPCR. The registry collected the data by active registration across the entire province and regular training by SEER staff. Data of all Gharbiah province patients diagnosed with uterine cancer and Gharbiah patients seeking medical advice outside the region were included in the registry.

Uterine cancers included in the registry were classified on the basis of the International Classification of Diseases codes (ICD-*O*-3).^19^ Corpus uteri included codes C54.0 to C54.9 and uterus not otherwise specified cancer included code C55.9.

The tumor morphology code, grade, and stage classifications were described previously in our previous study.^16^ However, a recent genomic classification for endometrial cancer may have implications for the prognosis and management of the disease.^20-22^ These classifications are polymerase epsilon ultramutated, microsatellite instability hypermutated, copy-number low, and copy-number high.^20^ Unfortunately, these data were not available because the genetic tests were not part of the cancer registry of the Gharbiah region. Tumor stages were grouped into four stages: local, regional, distal, and unstaged.

The GPCR adopted the SEER staging system, whereby localized cancer was considered stage 1; regional included stages II, III, and IVA cancers; distant included stage IVB cancers; and when the surgeon was unable to classify the tumor or the data were missing, this was considered unstaged.

Parity was grouped into three categories. Nulliparity was defined as women who have no children, the low-parity group consisted of those who have only one child, and the high-parity included those having two or more children.

Rural or urban residence was based on the classification of the Egyptian Central Agency for Public Mobilization and Statistics. The Gharbiah province consists of the following eight districts: Tanta, Mehalla, Kafr Zayat, Zefta, Samanood, Santa, Kotoor, and Basyoon. Each district has a capital city and surrounding villages. The population of the capital city of each district was considered urban, whereas the population of the remaining areas was considered rural.^17^ Residence was considered rural or urban on the basis of the permanent residence address of the patients.

The total population of women in Gharbiah province was 1,977,324 in 2006 (last published census).^17^ Approximately 70% of residents were rural, and 30% were urban. Data from the Egyptian census of 2006 were used for calculating IRs in this study.^17^ Medical care in the province is provided through a large network of public and private clinics and hospitals, including 257 primary health care units, 57 integrated hospitals, two central hospitals in rural areas, and 88 urban hospitals.

The IR of uterine cancer was calculated for the rural, urban, and total populations. The world standard population of WHO 2000 was used to calculate the age-standardized rate (ASR).^23^ The rate ratio was calculated to compare the rates in rural and urban areas and to examine the incidence trend over time. The study period was divided into three periods; 1999 to 2002, 2003 to 2006, and 2007 to 2010. The first period was used as the reference period for calculating the rate ratio; the rate in the last period was compared with the second period.

The crude IRs were calculated by dividing the total number of uterine cancers (E) by the total number of women at risk in the same population per 100,000. The formula of CI = IR ± 1.96 × IR/√E was used to calculate the 95% CI. The rate ratio of uterine cancer in urban versus rural areas was calculated by dividing the IR in urban areas (IR1) by the IR in rural areas (IR2). The CI of Rate Ratio = exp [In (IR1/IR2)] ± 1.96 × √(1/E1 + 1/E2). The χ^2^ test was used to compare categorical variables. A two-sided *P* value ≤ .05 was considered statistically significant. Statistical analysis was conducted using SAS version 9.4 (SAS Institute, Cary, NC). The study was approved by the institutional review board committees of the University of Nebraska Medical Center and the Gharbiah Cancer Society.

## RESULTS

Slightly more than one half of the patients (54%) resided in urban areas, and 46% of patients resided in rural areas. There were no significant differences between patients residing in rural and urban areas with respect to menopausal status (rural premenopausal [18%] *v* urban premenopausal [17%]) and parity (rural nulliparous [48%] *v* urban [49%]). However, occupation rates were significantly different in rural versus urban women; only 4% of rural women were employed compared with 19% of urban women (*P* < .001).

Tumor morphology had a significant association with residential status. Adenocarcinoma was the most common type in both rural and urban patients (66% and 70%, respectively). Sarcoma was found equally (11%) in each of the urban and rural patient groups. Approximately 8% of urban uterine cancers were classified as other carcinoma, whereas double that proportion (16%) was found in rural areas. The majority of uterine cancers were diagnosed as localized in both urban (56%) and rural (64%) areas. Approximately one half of the patients (48%) for whom treatment data were available had surgery alone, in both rural and urban areas. Tumor stage, grade, and treatment did not show any significant association with place of residence (Table 1).

**TABLE 1:**
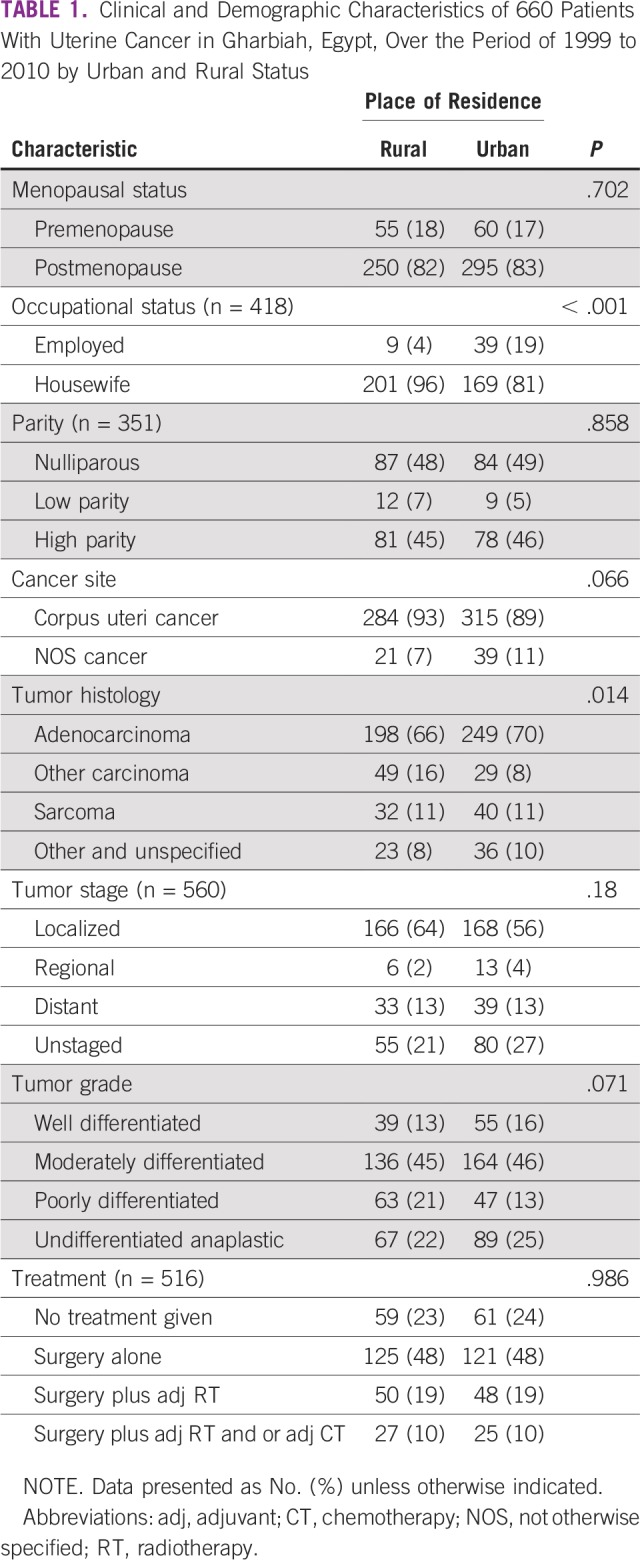
Clinical and Demographic Characteristics of 660 Patients With Uterine Cancer in Gharbiah, Egypt, Over the Period of 1999 to 2010 by Urban and Rural Status

Urban areas had the highest age-specific rates for all age groups (Table 2). However, rural and urban patients experienced the same pattern of increasing rates with an increase in women’s age, up to the age group of 60 to 69 years.

**TABLE 2:**
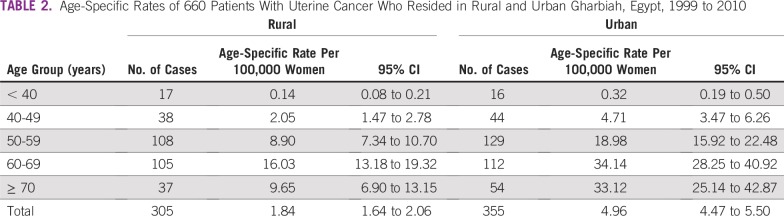
Age-Specific Rates of 660 Patients With Uterine Cancer Who Resided in Rural and Urban Gharbiah, Egypt, 1999 to 2010

The crude rates of uterine cancer were consistently higher in urban than in rural areas over the period of 1999 to 2010. The overall crude IR of uterine cancer over the period of 1999 to 2002 was 1.95 (95% CI, 1.64 to 2.25) per 100,000. The rural crude IR was 0.78 per 100,000, whereas the rate was 4.65 per 100,000 in urban areas. The gap between the two crude IRs of rural and urban areas declined in the last two periods (2003 to 2006 and 2007 to 2010).

The rural ASR was 2.8 (95% CI, 2.48 to 3.13) per 100,000 compared with 6.9 (95% CI, 6.13 to 7.66) per 100,000 in urban areas. The rate ratio showed that the IR of uterine cancer in urban areas was 2.46 times the rate in rural areas (Table 3).

**TABLE 3:**
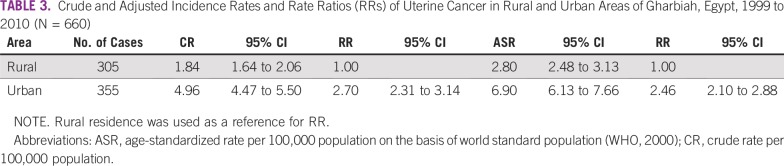
Crude and Adjusted Incidence Rates and Rate Ratios (RRs) of Uterine Cancer in Rural and Urban Areas of Gharbiah, Egypt, 1999 to 2010 (N = 660)

**FIG 1:**
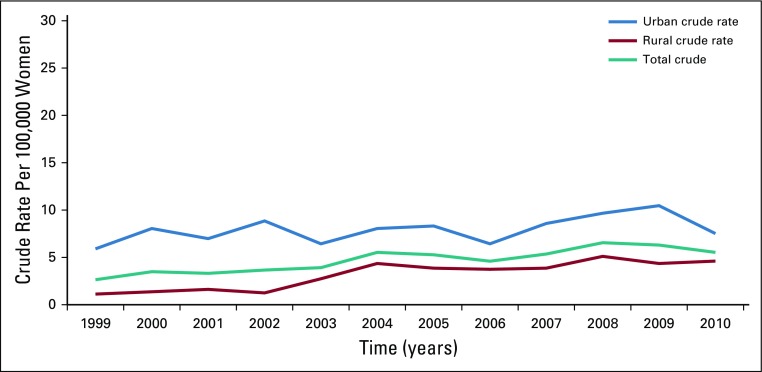
Time trend of uterine cancer crude rate in Gharbiah province, 1999 to 2010.

Table 4 lists the crude rate of uterine cancer over time for each of the geographic areas of Gharbiah. The crude rate increased over time in both rural and urban areas. The rate ratio between time periods in rural areas, with 1999 to 2002 as a reference period, increased steadily and significantly over time; the crude rate ratio was 2.7 (95% CI, 1.94 to 3.89) for 2003 to 2006 and 3.4 (95% CI, 2.38 to 4.71) for 2007 to 2010, in comparison with the reference time period. Although the crude rate was always higher in urban areas, the rate ratio did not show any significant increase over time. Figure 1 illustrates the steady increase in crude rates in both rural and urban areas over time.

**TABLE 4:**
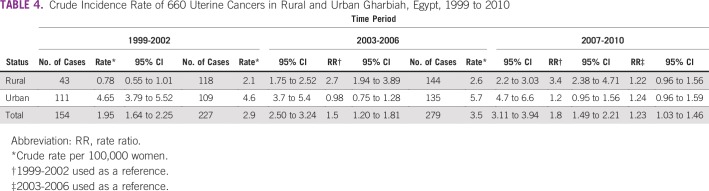
Crude Incidence Rate of 660 Uterine Cancers in Rural and Urban Gharbiah, Egypt, 1999 to 2010

## DISCUSSION

This study revealed the following interesting observations. First, there were no differences in most of the clinical and demographic characteristics of patients from rural and urban areas. Second, age-specific rates showed similar patterns of increase with aging for patients in both rural and urban areas. Third, the uterine cancer IR was significantly higher in urban areas than in rural areas. Fourth, uterine cancer IRs increased from 1999 to 2010 in rural areas but not in urban areas.

Regarding the lack of difference in clinical and demographic characteristics between patients from rural and urban areas and the increasing uterine cancer rates with aging, it is important to note that lack of accessibility and unaffordability of health care in these populations are not impediments to receiving appropriate cancer management. Health care facilities (health units, primary care centers, private clinics, and private and public hospitals) are available and accessible to both rural and urban populations. For example, more than 300 primary health care centers, integrated hospitals, and central hospitals are distributed throughout the rural areas. In addition, the distance between remote rural sites of the province and the respective capital cities of the districts is no more than 20 km. Furthermore, various methods of transportation are available and affordable if special diagnostic or treatment regimens are needed. It has been shown in the United States that increasing the accessibility to health care has resulted in a reduction of mortality rates by providing early diagnosis and specific treatment of uterine cancer.^24^ In addition, improving diagnostic and management facilities in Gharbiah showed a significant impact on diagnosing patients with cancer included in the Gharbiah registry in both areas.^18,25^

A systematic review of the literature from 1992 to 2012 that included a total of 366,299 patients with uterine cancer in the United States revealed an association between socioeconomic status (SES), incidence, stage at diagnosis, and survival rates.^26^ Patients with a low SES presented more frequently with advanced-stage uterine cancer, which may be attributed to these patients’ poor accessibility to health care.^27-29^ Disparities in access to health care may explain the relationship between SES and differences in IRs and stages of diagnosis of uterine cancer in the United States. However, health care accessibility and affordability were not barriers to receiving care in the rural and urban areas of our study population in Egypt. This may explain why tumor grades and stages at diagnosis were not associated with the place of residence in our study population in Egypt. The pattern and increment of age-specific rates among rural and urban women in this population were consistent with global trends for uterine cancer in both developed and developing countries, as reported in Cancer Incidence in Five Continents.^23^

The higher incidence of uterine cancer in urban than in rural areas was observed in the previous study.^13^ That study was based on the registry data of a shorter time period (1999 to 2001), and it showed crude IRs of 0.74 and 4.52 per 100,000 in rural and urban areas compared with 1.84 and 4.96 per 100,000 in rural and urban areas in this current study.^13^ The ratio of urban to rural rates decreased from 6.07 in the previous study to 2.70 in the current study. The changes in IRRs over the study period indicate that rural areas experienced an increase in IRRs, whereas the rates remained stable in urban areas; this variation was reflected in the narrowing gap between rural and urban rates. Although the rural ASR of uterine cancer in this study (2.8 per 100,000) is significantly lower than the urban ASR (6.9 per 100,000), it is relatively close to the global average ASR.^30^ The variation in the IR of cancer in rural and urban areas was observed previously in breast cancer. Similar to our study, that study observed a higher incidence of breast cancer in urban areas compared with rural areas of Gharbiah.^31^

The significant difference between rural and urban rates, as well as the increasing rural rates, revealed in this study might be a reflection of changes in risk factors of uterine cancer in this population over time. Examples of these risk factors include parity, obesity, early age of menarche, use of contraceptives, and physical activity.

Parity has been inversely associated with uterine cancer in several studies from various countries.^32-35^ In Egypt, higher fertility rates have been reported in rural than in urban areas, including in our study population in Gharbiah. It is important to note that fertility rates have declined in rural areas of Gharbiah, from 4.6 per 1,000 women in 1988 to 3.6 per 1,000 women in 2014, and from 3.8 per 1,000 women in 1988 to 3.0 per 1,000 women in 2014 in urban areas.^36^

Obesity is a strong risk factor for uterine cancer.^37-40^ The obesity and overweight data that were collected by the WHO showed that Egypt has a high obesity rate compared with many countries worldwide.^41^ During the 1990s, the average body mass index was higher in urban women (29.8 kg/m^2^) than in rural women (26.8 kg/m^2^).^42^ This gap declined over time. The average body mass index was 31.1 kg/m^2^ in urban areas and 31.0 kg/m^2^ in rural women in 2014. The risk factor of uterine cancer in rural women has increased compared with those in urban areas.

Early age of menarche has been identified as one of the factors that present a lifetime risk of uterine cancer.^43^ In Egypt in the 1980s, urban girls tended to have an earlier age of menarche compared with those in rural areas.^44^ However, age at first marriage in rural areas is younger than in urban areas. The median age at first marriage was 20 and 22.4 years in rural and urban women in Egypt, respectively.^36^ Approximately 10% of young women in the age group of 15 to 19 years had had at least one pregnancy.^36^ Therefore, urban women were exposed to a higher risk of uterine cancer for a longer time over their lifetime compared with rural women.

Oral contraceptive rates were low and with no clear difference among the rural and urban women of Egypt.^36^ However, previous studies have suggested that higher rates of uterine cancer in urban than in rural areas might be related to higher xenoestrogenic exposures in urban areas.^13^ The stable IR in urban areas compared with rates in rural areas might be related to nutritional and epidemiologic transition.^45^ The urbanization of rural areas, dietary factors, food sources, and epigenetic factors^46-48^ may lead to exposures to estrogenic compounds in both urban and rural populations.^49-51^

Physical activity plays a significant role in the risk reduction of uterine cancer by lowering the level of serum estradiol, which may help in reducing weight gain.^52-55^ The physical activity of rural women in Egypt is significantly higher than the activity of urban women. Occupational activities in farming and livestock breeding and less technologic household environments translate to a higher level of activity in rural areas compared with urban areas.^17^

This study has several strengths. The study period spans more than 12 years. The population-based nature of the study and the validated quality of the data add to its strength. Comparing the results with previously published research from the same region highlights the impacts of changes in lifestyle and demographic and environmental factors on the epidemiology of uterine cancer in this population.

However, the study also has limitations. Like other population-based registries, the Gharbiah registry did not include information about lifestyle and risk factors. The relatively small number of uterine cancer cases in this population was another limitation.

In summary, this study revealed the increasing rates of uterine cancer in rural areas and stable but much higher rates in urban areas in Gharbiah, Egypt. Future studies should elucidate the etiology of uterine cancer in both urban and rural populations in Gharbiah and other regions in Egypt. Future studies should also quantify the impact of nutritional and epidemiologic transitions on this population. Although no significant difference existed in the stage of presentation or type of treatment, future studies should examine the diagnostic methods, treatment outcomes, and uterine cancer-specific mortality in rural and urban regions.
